# A novel role for monosodium urate monohydrate crystals and gouty synovial fluids in monocyte migration in gout

**DOI:** 10.1186/ar3606

**Published:** 2012-02-09

**Authors:** M Asif Amin, Qiang Shu, Jonathon W Vargo, Jeffrey H Ruth, Takeo Isozaki, Solhee Lee, Alisa E Koch

**Affiliations:** 1 University of Michigan Medical School, Ann Arbor, MI, USA; 2 Department of Veteran's Affairs and University of Michigan, Ann Arbor, MI, 48109, USA

## Background

Gout is characterized by intra-articular deposition of monosodium urate monohydrate (MSU) crystals. The role of neutrophil influx in acute gouty arthritis is well established, while the contribution of monocytes (MNs) and their secreted inflammatory mediators is not. Here we demonstrate the role of MSU in MN migration.

## Materials and methods

To examine the role of MSU crystals in normal human peripheral blood (PB) MN migration, we performed MN chemotaxis in a modified Boyden chamber *in vitro *using either MSU crystals or gouty synovial fluids (SFs) as stimuli. To examine mechanisms of MN migration, we performed MN chemotaxis with MSU in the presence or absence of chemical signaling inhibitors. We determined the *in vivo *role of MSU crystals or gouty SFs in homing of dye-tagged MNs using normal human synovial tissue (ST)-severe combined immunodeficient (SCID) mouse chimeras. To investigate the contribution of MSU to production of leukocyte chemoattractants macrophage migration inhibitory factor (MIF) and epithelial neutrophil activating factor-78 (ENA-78/CXCL5), and the signaling molecules involved in secretion of these cytokines, we stimulated MNs with MSU crystals with or without chemical signaling inhibitors, and performed ELISAs on conditioned medium. We also assayed for MIF in gouty SF by ELISA.

## Results

We found a significant two fold increase in *in vitro *MN migration in response to MSU crystals, while gouty SFs increased MN migration five fold compared to negative control (p < 0.05). MSU crystal induced MN migration was significantly decreased by inhibitors of p38 MAPK, Src, and NFκB, suggesting that crystal induced MN migration occurs via these pathways. After engrafting SCID mice for 4 weeks, we injected dye-tagged human PB MNs via tail vein. Simultaneously, we injected MSU crystals or gouty SFs into ST grafts. After 48 hours, we harvested the STs and found an increase in MN homing to the grafts injected with MSU crystals or SFs (p < 0.05), indicating that either of these stimuli could recruit MNs *in vivo*. Human MNs stimulated with MSU for 24 hours released significantly higher quantities of the potent leukocyte chemoattractants MIF and ENA-78/CXCL5. MIF was six fold higher in gouty SFs compared to osteoarthritic fluids, suggesting the importance of MIF in gouty arthritis. MIF or ENA-78/CXCL5 secretion depended on the p38 MAPK pathway.

## Conclusions

This data suggests an intriguing role for MSU crystals and gouty SFs in MN migration and provides evidence that MNs and their secreted products may be potential therapeutic targets for treating gout.

